# An active piezoelectric plane X-ray focusing mirror with a linearly changing thickness

**DOI:** 10.1107/S1600577523009566

**Published:** 2024-01-01

**Authors:** Naxi Tian, Hui Jiang, Jianan Xie, Shuai Yan, Dongxu Liang, Zhisen Jiang

**Affiliations:** a Shanghai Synchrotron Radiation Facility, Shanghai Advanced Research Institute, Chinese Academy of Sciences, 239 Zhangheng Road, Pudong District, Shanghai 201204, People’s Republic of China; b Shanghai Institute of Applied Physics, Chinese Academy of Sciences, 2019 Jialuo Road, Jiading District, Shanghai 201800, People’s Republic of China; c ShanghaiTech University, 393 Middle Huaxia Road, Pudong District, Shanghai 201210, People’s Republic of China; Australian Synchrotron, Australia

**Keywords:** synchrotron radiation, piezoelectric deformable mirror, X-ray focusing, speckle metrology

## Abstract

X-ray mirrors for synchrotron radiation are often bent into a curved figure and work under grazing-incidence conditions due to the strong penetrating nature of X-rays to most materials. Mirrors of different cross sections have been recommended to reduce the mirror’s slope inaccuracy and clamping difficulty in order to overcome mechanical tolerances. With the development of hard X-ray focusing, it is difficult to meet the needs of focusing mirrors with small slope error with the existing mirror processing technology. Deformable mirrors are adaptive optics that can produce a flexible surface figure. A method of using a deformable mirror as a phase compensator is described to enhance the focusing performance of an X-ray mirror. This paper presents an active piezoelectric plane X-ray focusing mirror with a linearly changing thickness that has the ability of phase compensation while focusing X-rays. Benefiting from its special structural design, the mirror can realize flexible focusing at different focusing geometries using a single input driving voltage. A prototype was used to measure its performance under one-dimension and two-dimension conditions. The results prove that, even at a bending magnet beamline, the mirror can easily achieve a single-micrometre focusing without a complicated bending mechanism or high-precision surface processing. It is hoped that this kind of deformable mirror will have a wide and flexible application in the synchrotron radiation field.

## Abstract 2

An active piezoelectric plane X-ray focusing mirror with a linearly changing thickness is presented. Focusing performances of the prototype are measured and a single-micrometre focusing result is achieved.

## 1. Introduction

The new generation of synchrotron radiation facilities pursue high-flux focusing for coherent X-ray techniques such as coherent X-ray diffraction, X-ray photon correlation spectroscopy, X-ray ultrafast imaging, X-ray holography, *etc.* (Nugent, 2010 ▸; Shpyrko, 2014 ▸; Jensen *et al.*, 2012 ▸; Pfau & Eisebitt, 2016 ▸; Robinson & Harder, 2009 ▸). These new techniques will make it possible to conduct scientific research at ultra-high spatial resolution and ultra-fast time resolution in the future.

To meet different experimental requirements, X-ray focusing systems aim to be smarter, more stable and achieve an extremely small focal spot. Conventional mechanical bending devices, which can use adjustable bending forces at either end or other positions of the mirror to yield asymmetric bending moments (Eng *et al.*, 1998 ▸), have been widely used at a variety of beamlines. However, the complexity and instability of these mechanisms place very high requirements on the mirror surface shape. These negative effects include clamping bending, gravity release, mechanical shock, thermal deformation, *etc.*, which appreciably affect the wavefront propagation of the X-ray beams and the spatial resolution of the optical system. Fewer mechanical bending adjustments result in an imperfect surface shape, even creating additional figure errors. Various methods have been used to compensate for some of these effects. Mao optimized the width of the substrate to match the combined effects of gravitational and bending moments (Mao *et al.*, 2017 ▸), but the improvement in focusing was limited by the gravitational effect of the thin substrate. Howells designed metal mirrors for high-power X-rays with a varying cross section which simplified the clamping requirements of the mirror that had adjustable curvature. Through the analysis and optimized design, they proposed a way to minimize slope errors and reduce hinge-induced errors (Howells & Lunt, 1993 ▸; Howells, 1995 ▸). However, for new-generation light sources, the thermal load on front-end optics such as the monochromator obviously increases. In some extreme cases the power density of the first crystal is even greater than 50 W mm^−2^. When a high-thermal-load element works in conjunction with a cryogenic cooling system, the thermal deformation was observed to become complex and unstable (Rutishauser *et al.*, 2013 ▸; Jiang *et al.*, 2020 ▸). It is obviously difficult for such wavefront errors to be fully compensated by mechanical bending (Padmore *et al.*, 1996 ▸) or gravity correction (Mao *et al.*, 2017 ▸).

In this situation, active or adaptive reflective optics have been introduced into the focusing system (Mimura *et al.*, 2010 ▸; Matsuyama *et al.*, 2018 ▸). As an important member of active/adaptive optics, bimorph mirrors have been successfully applied in the fields of astronomy and laser processing (Madec, 2012 ▸; Salter & Booth, 2019 ▸). Benefiting from its mechanics-free, compact and relatively cost-effective features, scientists from the ESRF first introduced two bimorph mirrors to synchrotron radiation beams in the Kirkpatrick–Baez (KB) geometry and achieved a focused spot of 10 µm (vertical) × 20 µm (horizontal) (Susini *et al.*, 1996 ▸). Subsequently, they also developed a bimorph mirror with multi-segmented electrodes to compensate for the low-frequency figure errors of a long mirror (Signorato *et al.*, 1998 ▸). Until now, with the development of X-ray focusing and the commercialization of SESO and JTEC, bimorph mirrors have been widely applied at many synchrotron radiation and free-electron laser (FEL) sources around the world, including SPring-8 (Signorato *et al.*, 2001 ▸), DLS (Nistea *et al.*, 2019 ▸; Alcock *et al.*, 2023 ▸), APS (Shi *et al.*, 2022 ▸), ALS (Sanchez del Rio *et al.*, 2020 ▸), NSRL (Yuan *et al.*, 2023 ▸) and EU-FEL (Vannoni *et al.*, 2016 ▸). The high-precision manipulation ability enables the focusing element to obtain a high-precision surface figure with a larger numerical aperture, even without improving the polishing processing. However, bimorph mirrors, especially in X-ray grazing reflective optics systems, can realize sub-nanometre surface correction (Jiang *et al.*, 2019 ▸; Sutter *et al.*, 2019 ▸). Regarding this type of mirror, the local radius of curvature is proportional to the square of the mirror thickness. This feature makes it possible to use a mirror with a linearly changing thickness to directly realize a complicated quadratic surface, such as elliptical, parabolic cylinder or even toroidal surface using simple piezoelectric elements. In this paper, we present the theoretical design of this kind of active piezoelectric deformable mirror with a linearly changing thickness. We also propose a prototype to realize single-micrometre X-ray focusing at a bending magnet beamline.

## 2. Theory

**Figure 1:**
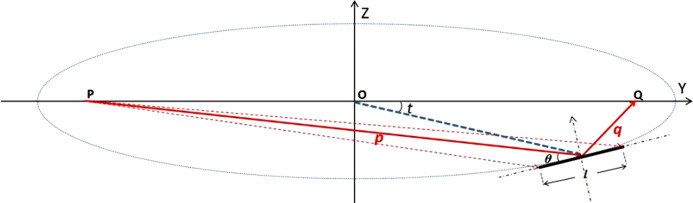
Geometric diagram of an elliptical focusing system. An incident beam from point *P* at an angle θ was focused to the point *Q* for an elliptical focusing system. The distance between *P* and the mirror center is the object distance *p*, and the distance from the mirror center to *Q* is the image distance *q*.

As can be seen in Fig. 1 ▸, a focusing mirror system satisfies the parametric equation of an ellipse,

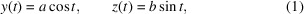

where *a* and *b* define the semi-major *Y* and semi-minor *Z* axes, and the parameter *t* is the central angle. The source *P* and the focus of the mirror *Q* are located at the two foci of the ellipse, and their coordinates are (−*c*, 0) and (*c*, 0) along the *Y* axis. The source-to-mirror distance is *p*, the focal length is *q*, and they satisfy the relationship *p* + *q* = 2*a*. The grazing-incidence angle of the mirror is θ. Regarding the mirror center, we use a subscript to distinguish *t* as *t*_0_. For any position of the ellipse, the local radius of curvature is presented by

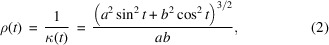

where κ is the curvature. The ideal mirror length can be regarded as the length of an arc with a small central angle interval, having the following relation at a given position,

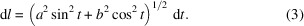

For a short mirror compared with a long source-to-mirror distance, the radius of curvature can be approximated by using a second-order Taylor series,

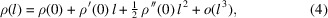

in which the coefficients are deduced to be

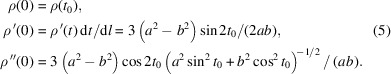

Here we approximate this elliptical shape by bending a flat mirror. Considering a bimorph active mirror, its radius of curvature obeys the relationship (Susini *et al.*, 1996 ▸)

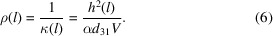

If it is assumed that the thickness of the mirror meets the linear variation of *h*(*l*) = *kl* + *m*, which is the most easily processed mirror type, equation (6) can also be transferred to a quadratic polynomial,

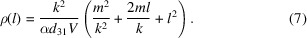

Comparing equations (4) and (7), it can be found that a perfect elliptical focusing mirror can be achieved in theory by bending a plane mirror with a linearly changing thickness when their corresponding coefficients are matched. For a specific applied voltage *V*, equation (7) includes two variables, *m* and *k*. There are three variables in equation (5), *a*, *b* and *t*, which can also be transferred to the three geometric parameters *p*, *q* and θ. Once we determine one of these three parameters, the other two parameters can be calculated according to the mirror parameters, or, based on the focusing requirement, the corresponding mirror parameters can be designed.

## 3. Prototype and experiments

**Figure 2:**
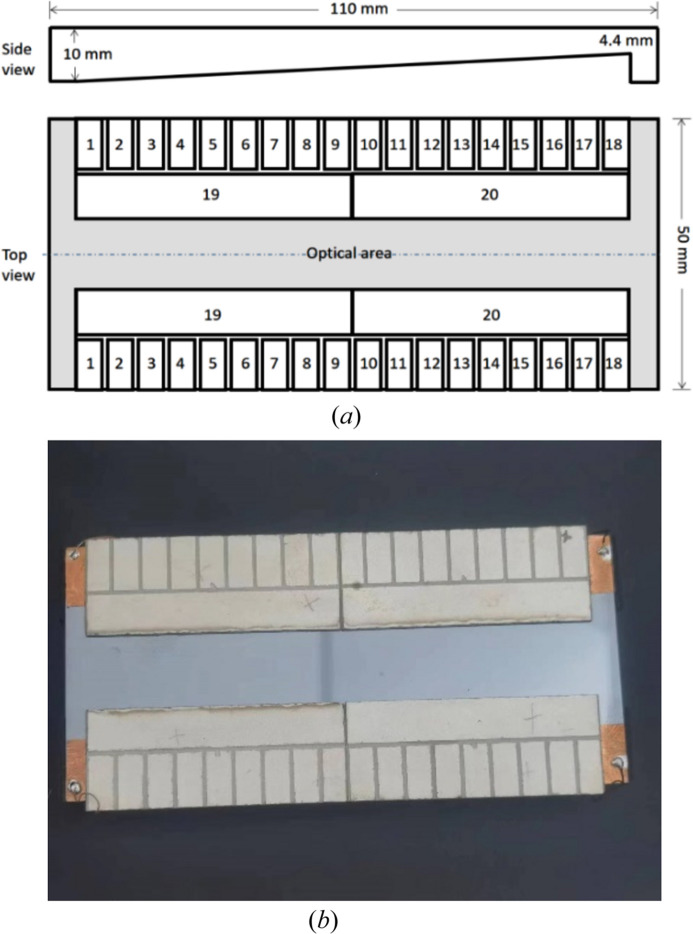
Diagram of the focusing mirror and arrangement of the piezoelectric ceramics. (*a*) The linearly changing thickness of the prototype silicon mirror is shown in a side view. Two long actuators (19 and 20) near the optical area are used to adjust the overall shape of the mirror to an ellipse. The small actuators 1–18 are used to adjust the overall shape and compensate the figure error of the elliptic surface to improve the focusing performance of this mirror. (*b*) Corresponding photograph of the prototype mirror.

As can be seen in Fig. 2 ▸, a prototype silicon mirror was made with a linearly changing thickness from 4.4 mm to 10 mm. The central stripe of the polished silicon mirror surface was regarded as the optical area. The piezoelectric effect of the material lead zirconium titanate was used to generate a global or local concave or convex shape. Two pairs of long piezoelectric elements were glued to the surface along the long sides and respectively bent by the actuators 19 and 20. The other 18 pairs of small piezoelectric elements were pasted outside of the long piezoelectric elements. Each pair of actuators was driven by an independent channel of a high-voltage power supply. The applied voltage ranged from −500 to +500 V.

**Figure 3:**
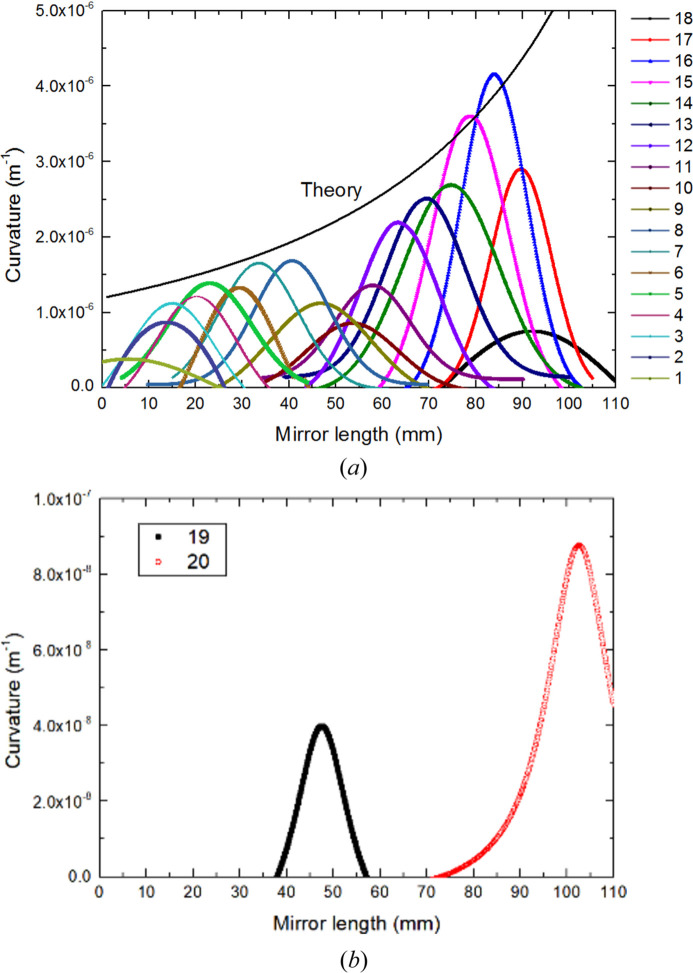
Measured piezoelectric response function (curvature per volt) of the prototype for actuators 1–18 (*a*) and actuators 19 and 20 (*b*). Because of the linearly changing thickness of the mirror, PRFs in thin areas are greater than those in thick areas.

The centers of the piezoelectric response functions (PRFs) almost change linearly with an increase in sequence number of the small actuators. As seen in Fig. 3 ▸(*a*), except for the two actuators at the two ends, the full widths at half-maximum (FWHMs) of the PRFs are 16.9 ± 2.6 mm, approximately coupled to cover three small actuators. As the thickness decreases, the PRF of the mirror increases significantly, and its tendency basically meets the theoretical relationship described in equation (6) which is plotted in Fig. 3 ▸(*a*). It is worth noting that the PRFs unexpectedly decrease near the mirror center. This phenomenon may result from the gap between the two glued pairs of long piezoelectric ceramics, but there is no clamping or support point in the middle of the mirror [as shown in Fig. 2 ▸(*b*)]. Another reason is that the mirror has an uneven distribution of gravity caused by the linear thickness change of the mirror. This situation is not the usual junction effect (Alcock *et al.*, 2013 ▸) and the junction effect has been eliminated in the new design of bimorph mirrors by changing the bonding mode of the piezoelectric ceramics (Alcock *et al.*, 2015 ▸). Each pair of long actuators acts on the half mirror. Due to the linearly changing thickness, the curvature change is mainly reflected in the thinner area, as seen in Fig. 3 ▸(*b*).

**Figure 4:**
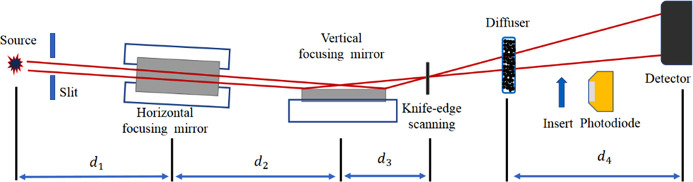
Optical schematic diagram of the experiment. The incident beam from a bending magnet source passes through the slit, HFM, VFM and then reaches the detector. HFM is the designed deformable mirror with linearly changing thickness.

**Figure 5:**
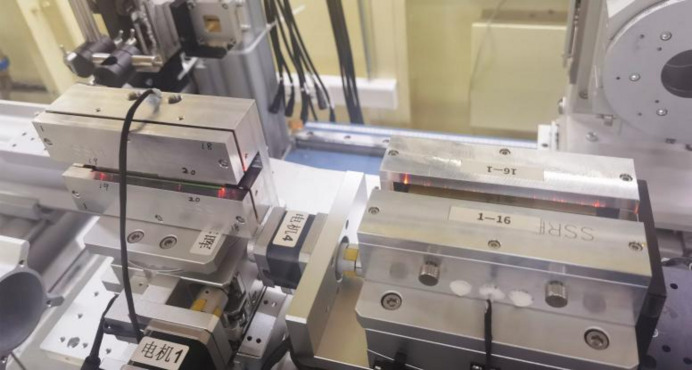
Photograph of the KB focusing system. The left HFM is the deformable mirror with linearly changing thickness and the right mirror is the VFM with a fixed elliptical shape.

The experiments were performed at the test beamline (BL09B) of the Shanghai Synchrotron Radiation Facility (SSRF) (Li *et al.*, 2019 ▸). As seen in Fig. 4 ▸, which shows an optical schematic diagram, the bending magnet source size is 155 µm × 55 µm, and a slit was used to limit the divergence. The photon energy of the incident beams was 9 keV to yield a high reflectivity over the whole mirror range with the material of silicon at a central grazing-incidence angle of 2.3 mrad. The KB focusing system shown in Fig. 5 ▸ was placed *d*_1_ = 39 m downstream of the source. The vertical focusing mirror (VFM) is a fixed-shape elliptic mirror, and the horizontal focusing mirror (HFM) is the deformable mirror mentioned above. The distance between the two mirror centers was *d*_2_ = 215 mm. A silicon slice at the focus *d*_3_ = 75 mm downstream of the HFM was used for a knife-edge test of the intensity profile with a scanning resolution of 25 nm. The silicon slice has a rectangular geometrical shape, making the sharp edge of the slice a simple and practical knife-edge. A photodiode (PD) was inserted into the reflected beam to record the intensity in the knife-edge test. A diffuser was located behind the knife edge and was used as a wavefront marker in speckle scanning metrology (Berujon *et al.*, 2012 ▸) for the figure correction of the deformable mirror. The piezo motion stage of the diffuser guaranteed a scanning step of 200 nm in the speckle scanning measurements. During the speckle scanning metrology, the deformable mirror was put as the vertical deflector for better spatial resolution. In this situation, the focal length is *d*_2_ + *d*_3_ = 230 mm. A microscope objective lens system (Optique Peter) coupled to a CMOS camera (Hamamatsu) was placed *d*_4_ = 825 mm downstream of the diffuser to acquire the speckle pattern. The effective pixel was *p* = 0.65 µm, and the exposure time was 10 s for each capture.

## 4. Results and discussions

**Figure 6:**
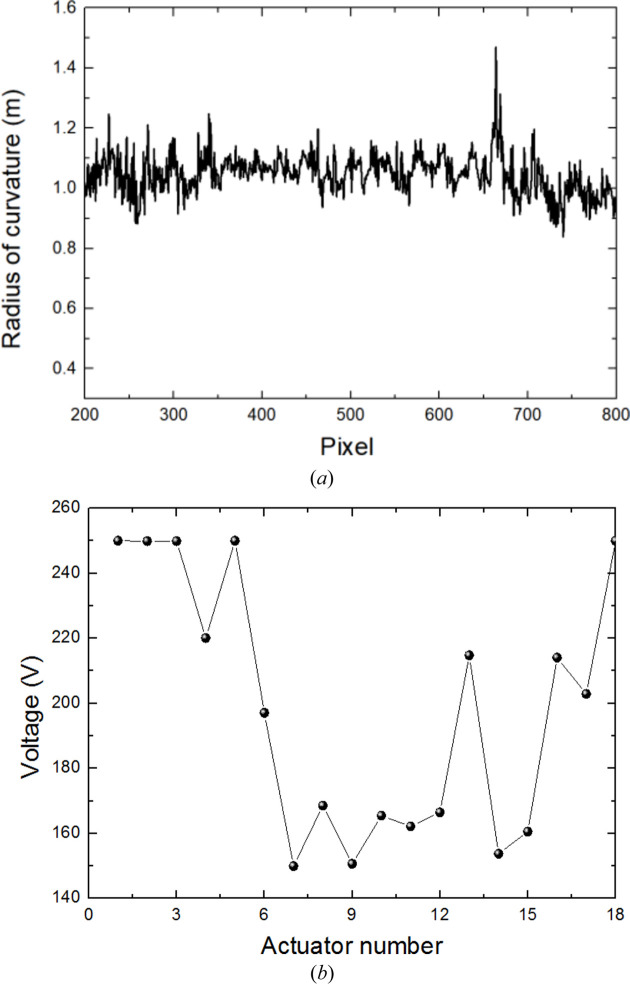
The final wavefront radius of curvature (*a*) of the designed deformation mirror measured from speckle scanning metrology after voltage compensation. (*b*) The applied voltages of 18 small actuators are calculated according to the radii of curvature of the deformation mirror which were measured by using speckle scanning metrology.

*In situ* speckle scanning metrology, based on measurement of the wavefront radius of curvature, was developed by Wang *et al.* (2015 ▸). After modulation by the deformation mirror, the wavefront arrives at the detector plane. The outgoing wavefront behind the deformation mirror was associated with the mirror figure error by the local wavefront curvature. The local wavefront curvature at the detector plane was acquired by the *in situ* speckle scanning technique. A series of speckle patterns were collected, and the digital image correlation (DIC) method (Pan *et al.*, 2009 ▸) was used to find the speckle displacement between two images that were stitched by these speckle patterns. Based on the speckle displacement calculated by DIC, the local wavefront curvature can be written as the following relation (Wang *et al.*, 2015 ▸), 

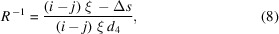

where *i* and *j* are two different rows of the speckle pattern, ξ is the effective pixel size of the detector and Δ*s* is the speckle displacement. The influence of vibration on this technique was discussed in the previous study (Tian *et al.*, 2022 ▸). Based on the wavefront radius of curvature acquired, the voltages for the different actuators were calculated using the PRF of the deformation mirror. After voltage compensation, the wavefront radii of curvature are shown in Fig. 6 ▸. The high-frequency oscillation of the radius curve may result from motor vibration or a low signal-to-noise ratio. However, this high-frequency oscillation has little effect on the focusing performance. Low- and medium-frequency wavefront curvature errors, which have a greater impact on focusing, were well suppressed. In order to verify the compensation results after applying input voltages, inverse Fresnel diffraction-based mutual intensity propagation was used to reconstruct the intensity distribution near the focal spot. The intensity can be expressed as

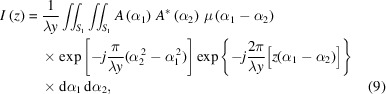

where the degree of coherence is μ(α) = 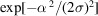 , where σ is the coherence length.

**Figure 7:**
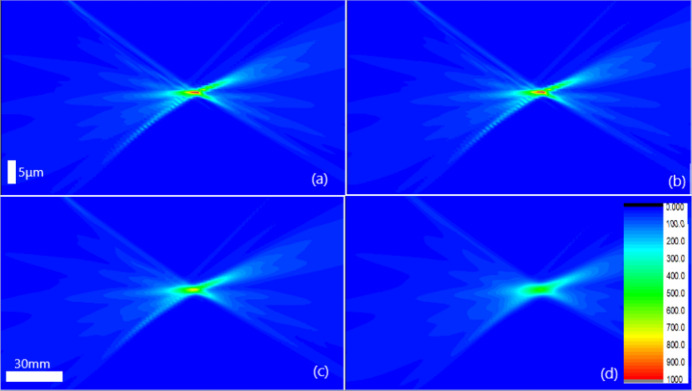
The intensity distribution near the focus reconstructed by inverse Fresnel diffraction based on the measured radius of curvatures for different degrees of coherence: (*a*) full coherence, (*b*) 10%, (*c*) 5% and (*d*) 3%.

**Figure 8:**
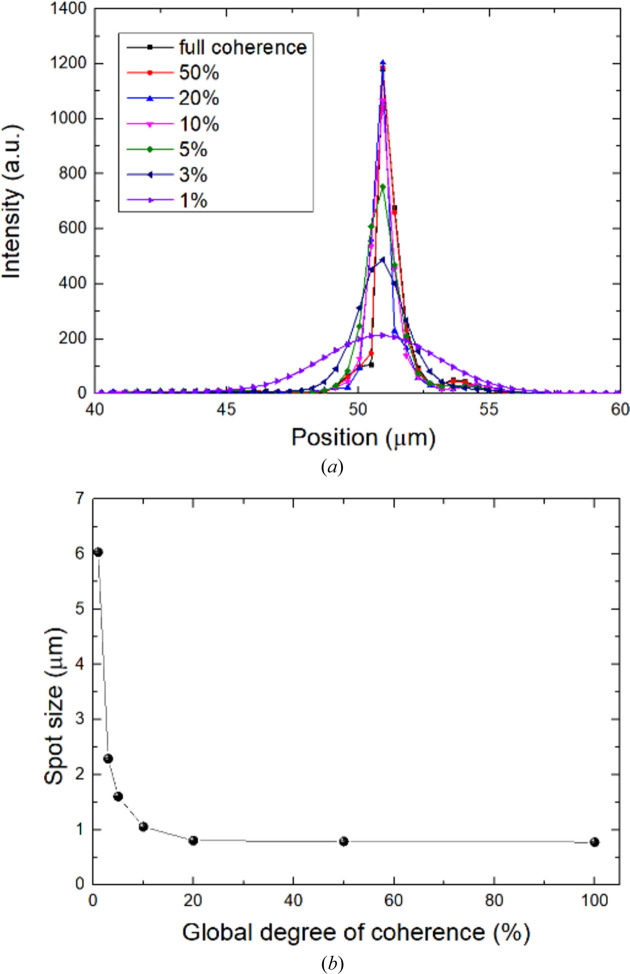
(*a*) Intensity profile of focal spots and (*b*) comparison of spot sizes with different global degrees of coherence.

As a bending magnet beamline, the degrees of coherence of BL09B for the vertical and horizontal directions are of the order of one-hundredth and one-thousandth, respectively. Fig. 7 ▸ shows the reconstructed intensity distribution near the focus for different degrees of coherence, and Fig. 8 ▸ compares the intensity profiles at the focus and their FWHM with the degree of coherence. For a degree of coherence of 3%, the spot size is ∼2.3 µm. If the degree of coherence is better than 20%, the spot size may be smaller than 800 nm.

**Figure 9:**
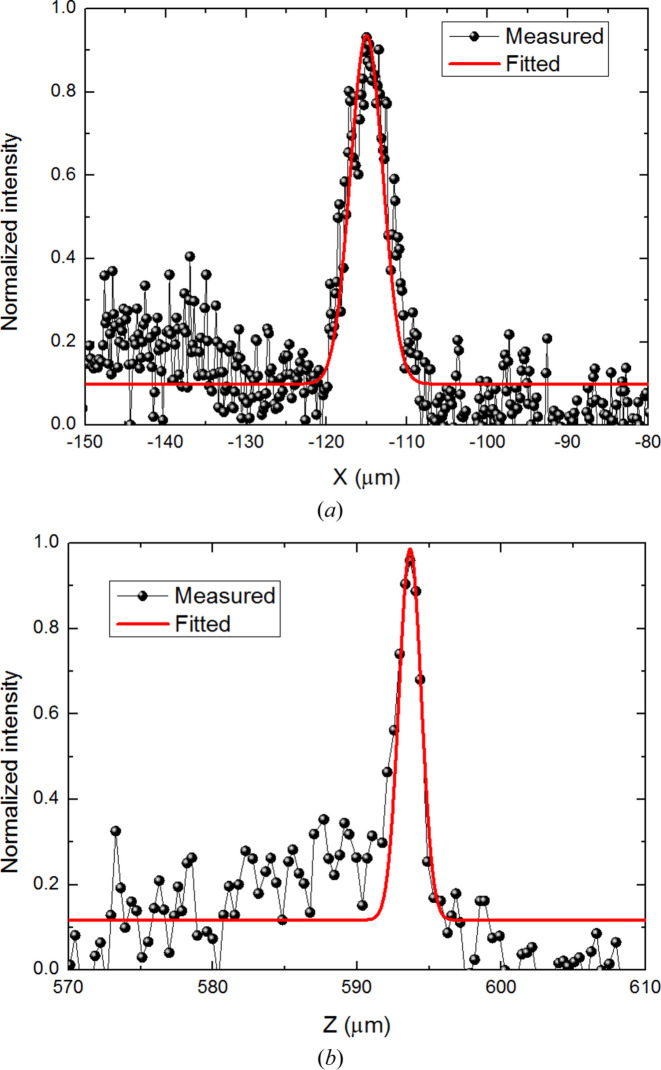
Profiles at the focus spot of the KB focusing system acquired by knife-edge scans in the (*a*) horizontal and (*b*) vertical directions after compensation.

Then we combined this deformable mirror and a fixed-shape elliptic mirror as a KB focusing system. The focal length of the deformable mirror increased from 230 mm to 290 mm. A knife-edge scan was used to measure the two-dimensional (2D) focus spot size of the focusing system. The intensity variation with a knife-edge scan was detected by the PD detector, and the focus spot size was derived by calculating the FWHM of the derivation curve from the recorded intensity. Both horizontal and vertical directions were scanned by knife edge, and the final focus spot size was 4.7 µm × 1.8 µm after compensation. The intensity profiles at the focal plane are shown in Fig. 9 ▸. Based on the compression ratio, the theoretical horizontal spot size is 1.2 µm. The focusing results show that an active piezoelectric plane mirror with a linearly changing thickness has the potential to realize satisfying X-ray focusing compared with a conventional mechanical bending mirror.

The slope error of a deformable mirror is an important problem for our KB focusing system, which widens the focus spot and thus limits the final focusing performance. Because of the inherent surface figure error generated in optical manufacture, the slope errors between the actual figure and the ideal figure are unavoidable for deformable mirrors. Although the surface shape of a deformable mirror is formed by different actuators into an elliptic shape, it cannot be a true ideal elliptic and cannot perfectly compensate for the total surface shape error of a deformable mirror. Therefore, the next step of our work is to enhance the focusing performance of the deformable mirror by decreasing the surface error of the deformable mirror in the processing stage and improving the surface control accuracy by optimizing piezoelectric elements and the corresponding control algorithm. The recently proposed closed-loop control method for deformable mirrors will also be the focus of our future work in mirror control. This method can quickly switch between different X-ray wavefronts without blocking or attenuating the X-ray beam. Combined with machine learning, the deformable mirror shape control algorithm based on data-driven modeling is also attractive, and can achieve surface shape precision control close to the X-ray diffraction limit (Gunjala *et al.*, 2023 ▸) and high-precision beam alignment (Xie *et al.*, 2023 ▸). Therefore, we will continue to optimize the design of our deformable mirror in future work by improving the machining accuracy, optimizing the structural design and the control algorithm to further improve its focusing performance. In this experiment, the insufficient beam flux of the bending magnet beamline leads to a weak signal-to-noise ratio; future similar experiments will perform better on undulator beamlines or even on fully coherent beamlines to significantly improve its focusing performances. We hope that the mirror we designed has the potential to be used in the next generation of synchrotron radiation sources and FELs.

## 5. Conclusion

Bimorph mirrors have been widely used in the X-ray focusing field at various synchrotron radiation sources around the world. Different from previous elastic bending mirrors at achieving an elliptical figure from a flat figure, we present an active piezoelectric deformable mirror with a linearly changing thickness to realize smart X-ray focusing without a complicated bending mechanism. A theoretical relationship between the mirror thickness change and focusing geometry is presented. A mirror with a linearly changing thickness can satisfy different requirements for the focal length. A prototype of the deformable mirror was fabricated and 1D and 2D focusing experiments were performed. Knife-edge scan and wavefront reconstruction methods both proved that the mirror can easily realize single-micrometre focusing with a long focal length, even at a bending magnet beamline.
